# How do healthcare providers respond to multiple funding flows? A conceptual framework and options to align them

**DOI:** 10.1093/heapol/czab003

**Published:** 2021-05-05

**Authors:** Edwine Barasa, Inke Mathauer, Evelyn Kabia, Nkoli Ezumah, Rahab Mbau, Ayako Honda, Fahdi Dkhimi, Obinna Onwujekwe, Hoang Thi Phuong, Kara Hanson

**Affiliations:** Health Economics Research Unit, KEMRI-Wellcome Trust Research Programme, Nairobi, Kenya; Department of Health Systems Governance and Financing, World Health Organization, Geneva, Switzerland; Health Economics Research Unit, KEMRI-Wellcome Trust Research Programme, Nairobi, Kenya; Health Policy Research Group, College of Medicine, University of Nigeria, Enugu Campus, Enugu, Nigeria; Health Economics Research Unit, KEMRI-Wellcome Trust Research Programme, Nairobi, Kenya; Research Center for Health Policy and Economics at the Hitotsubashi Institute for Advanced Study, Hitotsubashi University, Japan; Department of Health Systems Governance and Financing, World Health Organization, Geneva, Switzerland; Health Policy Research Group, College of Medicine, University of Nigeria, Enugu Campus, Enugu, Nigeria; Health Strategy and Policy Institute, Ministry of Health, Hanoi, Vietnam; Department of Global Health and Development, London School of Hygiene and Tropical Medicine, London, UK

**Keywords:** Provider payment methods, multiple funding flows, purchasing, provider behaviour

## Abstract

Provider payment methods are a key health policy lever because they influence healthcare provider behaviour and affect health system objectives, such as efficiency, equity, financial protection and quality. Previous research focused on analysing individual provider payment methods in isolation, or on the actions of individual purchasers. However, purchasers typically use a mix of provider payment methods to pay healthcare providers and most health systems are fragmented with multiple purchasers. From a health provider perspective, these different payments are experienced as multiple funding flows which together send a complex set of signals about where they should focus their effort. In this article, we argue that there is a need to expand the analysis of provider payment methods to include an analysis of the interactions of multiple funding flows and the combined effect of their incentives on the provision of healthcare services. The purpose of the article is to highlight the importance of multiple funding flows to health facilities and present a conceptual framework to guide their analysis. The framework hypothesizes that when healthcare providers receive multiple funding flows, they may find certain funding flows more favourable than others based on how these funding flows compare to each other on a range of attributes. This creates a set of incentives, and consequently, healthcare providers may alter their behaviour in three ways: resource shifting, service shifting and cost shifting. We describe these behaviours and how they may affect health system objectives. Our analysis underlines the need to align the incentives generated by multiple funding flows. To achieve this, we propose three policy strategies that relate to the governance of healthcare purchasing: reducing the fragmentation of health financing arrangements to decrease the number of multiple purchaser arrangements and funding flows; harmonizing signals from multiple funding flows; and constraining providers from responding to undesirable incentives.

**KEY MESSAGES**

- The article presents a conceptual framework that helps to assess multiple funding flows to providers.
- It is critical to understand the effects of multiple funding flows to health facilities, as the set of incentives they create affects provider behaviour and thus ultimately health system objectives.
- Multiple funding flows need to be aligned to set coherent incentives.
- Three strategies relating to the governance of purchasing are proposed: reducing health financing fragmentation to lower the number of multiple funding flows; harmonizing signals from multiple funding flows; and constraining healthcare providers to respond to undesirable incentives.

## Introduction

Health financing has three core functions, namely revenue raising, pooling and purchasing of health services (WHO, 2010). While health financing reforms have hitherto focused on resource generation and pooling of funds, increasing attention is now also given to the healthcare purchasing function (Mathauer *et al.*, 2017; WHO, 2017; Hanson *et al.*, 2019; Sanderson *et al.*, 2019). Purchasing refers to the allocation of resources from a purchasing agent to a healthcare provider in exchange for providing health services (WHO, 2010). It involves three sets of decisions: what to purchase (specifying benefit packages/service entitlements), whom to purchase from (specifying healthcare facilities), and how to purchase (provider payment methods and contractual arrangements between purchasers and healthcare providers) (WHO, 2010; RESYST, 2014).

Purchasing is undertaken within a healthcare market by various purchasing actors, such as the Ministry of Health, a social or national health insurance agency, (for-profit) private health insurance schemes, community-based health insurance funds, or non-governmental organizations (WHO, 2010). While individuals also pay providers directly through out-of-pocket expenditure, they do not interact and negotiate with providers in the same way that purchasing agencies can (Smith *et al.*, 2005).

Purchasing can be passive or strategic. Purchasing is passive when it involves merely paying bills or allocating historically based budgets, while strategic purchasing implies active, evidence-based engagement across the three purchasing decisions to pursue health system objectives (WHO, 2010). Strategic purchasing plays a critical role in achieving health system objectives of efficiency, equity, financial protection and quality, as such contributing to progress towards universal health coverage (Mathauer *et al.*, 2019a). It entails strategically shaping the interwoven relationships between the purchaser, the citizens, health service providers and the government (RESYST, 2014), thereby also reducing health care market failures of asymmetric information between individuals (patients) and providers (Preker *et al.*, 2007).

While provider behaviour is influenced by a wide range of policies and organizational arrangements, a key policy instrument for strategic purchasing is how healthcare providers are paid by purchasers (provider payment methods). Table 1 outlines the main provider payment methods (PPMs). PPMs are important policy levers because they generate signals that may influence healthcare provider behaviour in ways that may impact on the health system objectives (Langenbrunner *et al.*, 2005).

**Table 1:** Main payment methods used in health systems and expected incentives

Payment method	Definition	Likely incentives when existing or analysing in isolation without considering funding flow attributes
Prospective:		
Line-item budget	Providers receive a fixed amount to cover specific input expenses (e.g. staff, medicines), with limited flexibility to move funds across these budget lines	Under-provision, no focus on quality or outputs unless specified and held accountable
Global budget	Providers receive a fixed amount of funds for a certain period to cover aggregate expenditures. The budget is flexible and is not tied to line items.	Under-provision, also in terms of quality or outputs unless specified and held accountable; more potential for efficiency due to budget flexibility
Capitation	Providers are paid a fixed amount in advance to provide a defined set of services for each person enrolled for a fixed period of time.	Under-provision, over-referral (if unit of payment does not include some referral services)
Retrospective:		
Fee-for-service	Providers are paid for each individual service provided. Fees are fixed in advance for each service or group of services.	Increased provision, or over-provision
Case-based (or diagnosis related groups)	Hospitals are paid a fixed amount per admission depending on patient and clinical characteristics.	Increase of volume, reduction of costs per case, avoidance of severe cases
Per diem	Hospitals are paid a fixed amount per day so that an admitted patient is treated in the hospital.	Extended length of stay, reduced cost per day; cream-skimming

*Source*: Adapted from Cashin (2015).

Previous research has tended to focus on conceptualizing and analysing individual provider payment methods in isolation (Kazungu *et al.*, 2018). Research on strategic purchasing has also tended to adopt the perspective of the purchaser to describe the purchasing arrangements they apply in their relationships with providers (e.g. Honda *et al.*, 2016; Etiaba *et al.*, 2018; Patcharanarumol *et al.*, 2018). However, in practice, purchasers typically use a range of PPMs to pay healthcare providers for different types of services. Further, low- and middle-income countries often have fragmented health financing systems that are characterized by multiple purchasers; and even health systems with a single purchaser are often characterized by the existence of multiple payment methods, such as for inpatient care, outpatient care or national programmes for specific diseases.

As a result of this fragmentation of health financing arrangements, healthcare facilities in most low- and middle-income countries are paid based on multiple payment methods and receive funding from multiple purchasers. This constitutes a mixed provider payment system (Mathauer *et al.*, 2017; Mathauer *et al.*, 2019a).

From a healthcare provider perspective, these multiple payments from multiple purchasers represent multiple funding flows to them. While understanding the incentives generated by individual PPMs is an important starting point, there is a need to expand this focus to analyse the interactions of multiple funding flows and the combined effect of their inherent incentives from the perspective of the healthcare provider (Mathauer and Dkhimi, 2019).

To seize the complex nature of the financial relationship between purchasers and healthcare providers, in this paper we conceptualize this relationship more comprehensively and capture it under the term *multiple funding flows*. We define a funding flow as any transfer of resources, in cash or in kind (e.g. including the deployment of health workers, supply of medicines and medical goods) from a purchaser to a healthcare provider for the provision of healthcare services. In this perspective, out-of-pocket payments from an individual to a healthcare provider are also included in the analysis of funding flows, even though they are not an organized purchaser. We use the term healthcare provider here to refer to the organizations that provide healthcare services (e.g. hospitals, health centres, clinics), rather than individual healthcare workers (e.g. doctors and nurses).

A funding flow is characterized by a distinct combination of features. These features are:

- Services to be purchased
- Group of patients or population group to be covered
- Provider payment method to be used
- Provider payment rate used
- Accountability mechanism to be used

The various funding flows going to healthcare providers should ideally be coherent; that is, they should generate incentives that are complementary (rather than conflicting) and aligned to health system goals. However, too often they are non-aligned, sending contradictory signals to healthcare providers, with the likely effect of distorting their behaviour (WHO, 2017).

The objectives of this article are to highlight the importance of multiple funding flows to health facilities by characterizing the phenomenon from the healthcare provider perspective and to develop and present a conceptual framework for analysing how multiple funding flows create a set of incentives that influence healthcare provider behaviour, and in turn may affect health system objectives.

The methods section outlines how this conceptual framework was developed. In the results section, we then present this conceptual framework in detail, followed by a reflection on the methodological challenges of assessing multiple funding flows. A section on policy options for managing multiple funding flows reflects on governance arrangements to address their consequences. A conclusion is provided in the last section.

## Methods

This section outlines how we developed the proposed conceptual framework. It was elaborated jointly by the authors of this article, who were involved in two related programmes of work: a study of purchasing arrangements conducted by the RESYST consortium (RESYST, 2014; Ibe *et al.*, 2017; Etiaba *et al.*, 2018; Patcharanarumol *et al.*, 2018; Hanson *et al.*, 2019; Mbau *et al.*, 2020) and WHO’s global conceptual and country work on strategic purchasing and mixed payment systems (Mathauer *et al.*, 2017; WHO, 2017; Feldhaus and Mathauer, 2018; Mathauer and Dkhimi, 2019, WHO, 2019). Both teams identified the issue of mixed payment systems and multiple funding flows as an understudied issue and conducted country case studies to describe them and their effects (Appaix *et al.*, 2017; Mbau *et al.*, 2018; Phuong *et al.*, 2018; Mathauer *et al.* 2019b; Jaouadi *et al.*, 2020; Onwujekwe *et al.*, 2020). The work was also informed by the teams’ knowledge and work/research experience at country level, harnessed through brainstorming sessions. Each team had developed their study approach, shared and presented these at technical meetings and conferences and both teams had reviewed each other’s approach. After the country case studies were completed, and in view of the similar findings, the teams met at a face-to-face workshop to harmonize and refine their approaches and develop this conceptual framework. The teams also performed a cross-case analysis, which will be published elsewhere.

## Results: the conceptual framework

**Figure 1:**
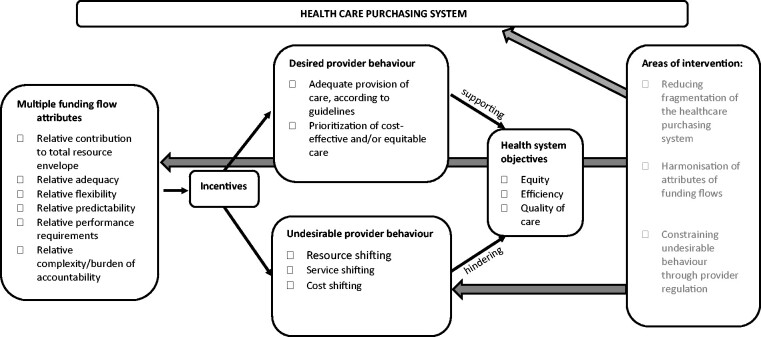
Conceptual framework of multiple funding flows. *Source*: Authors.

The framework explains how multiple funding flows to a healthcare provider, together with their attributes, create a set of incentives that influence provider behaviour, thus affecting health system objectives. The sub-sections below explain each of these aspects. Figure 1 below outlines the framework.

### Multiple funding flows and attributes

We hypothesize that when healthcare providers receive multiple funding flows, they may find certain funding flows more favourable than others based on how these funding flows compare to each other on a range of attributes. These attributes are:

1. The contribution that each funding flow makes to the total healthcare provider income
2. The adequacy or sufficiency of each of the payment rates to cover the costs of services purchased
3. The level of managerial flexibility and financial autonomy that healthcare providers have over each of the funding flows
4. The complexity and burden of accountability mechanisms associated with each of the funding flows
5. The predictability in terms of timing of disbursement and amounts of each of the funding flows
6. The performance requirements of funding flows, when these are linked to remuneration or sanctions.

### Set of incentives and their influence on provider behaviour

We further hypothesize that when healthcare providers are faced with multiple funding flows that vary according to one or more of these attributes, they may alter their behaviour depending on how favourable they find certain funding flows over others. The incentives which characterise these preferred funding flow(s) will dominate and influence a provider behaviour response. These behavioural responses due to multiple funding flows need to be analytically distinguished from provider behaviour that is directly caused by incentives inherent in an individual payment method, such as DRG upcoding or cream skimming under capitation. They will also be mediated by the firm’s objectives (e.g. profit maximization versus certain cultural or other values that would make them favour some payers over others), by organizational structures (e.g. the extent of vertical integration), and by the contractual arrangements through which individual health workers are linked to the organization (e.g. how doctors are paid and whether they are allowed to engage in dual practice).

In the ideal case, incentives of funding flows are complementary and compensatory to each other and create incentives for provider behaviour to contribute to efficient and equitable quality service provision (Barnum *et al.* 1995; Langenbrunner *et al.* 2009). However, the provider behaviour resulting from the incentives generated by multiple funding flows may undermine the achievement of the health systems objectives mentioned above (Mathauer and Dkhimi 2019). The following three provider behaviours that may be either desired or undesired in certain contexts are likely to result from (incoherent) incentives generated by multiple funding flows, particularly when the funding flow(s) under question come along with weak accountability mechanisms:

#### Resource shifting

This occurs when healthcare providers preferentially shift *resources* in order to provide services under a particular funding flow. For example, healthcare providers could allocate more beds, more nurses and/or doctors or their time, and or more essential supplies to a specific set of services, specific hospital departments or wards used by patients who are covered by a more favourable funding flow.

#### Service shifting

This occurs when a healthcare provider shifts *service provision* under a less favourable funding flow to a more favourable one. For example, where publicly funded laboratory services as well as commercialized, privately funded laboratory services exist within the same healthcare facility, patients that are perceived to be able to pay out-of-pocket could be directed from the former to the latter where higher user charges are raised. Or, instead of treating a patient in the outpatient department, healthcare providers may shift the patient to its inpatient care department and thus unnecessarily admit a patient because they consider inpatient payment methods and rates more favourable compared to payment rates in the outpatient department. They may also unnecessarily discharge patients early to attend to them at the outpatient department if the converse is true. Patients that are covered by a health insurance scheme could be asked to additionally pay out-of-pocket because the insurer’s reimbursement rates are considered inadequate.

#### Cost shifting

This occurs when healthcare providers shift *costs* by charging higher rates for the same service to one funding flow, so as to compensate for a lower payment from another funding flow or when another payment flow goes down (relative to costs or trends). As such, one overpays, whereas another one underpays relatively. Or healthcare providers might charge higher rates to patients with health insurance coverage and lower rates to cash paying users for the same service, since cash paying users may be unable to pay the amounts charged to health insurance schemes. In this case, the healthcare provider would engage in price discrimination (Frakt, 2011).

Healthcare providers are likely to respond to the mixed set of signals from multiple funding flows by engaging in multiple behaviours concurrently; and may even do so at quite a disaggregated level, e.g., service by service. This means that from an analytical perspective it may be difficult to distinguish individual responses, and from a regulator’s perspective, it may be difficult to align incentives with health system goals.

### Potential influence of provider behaviour on health system objectives

The behaviour incentivized by the attributes of multiple funding flows could influence health system objectives, as outlined in Table 2. While there are many other factors influencing the achievement of health system objectives, such as health financing policy design and implementation, service delivery models, health worker skills, overall government health expenditure, etc., we focus on the effect of provider behaviour. The table looks at the effects of each multiple funding flow attribute on provider behaviour in isolation, although in practice, a healthcare provider may respond to the combination of attributes within one funding flow.

**Table 2:** Potential influences of multiple funding flows on provider behaviour and potential negative outcomes

Attributes of multiple funding flow	Potential provider behaviour	Potential negative outcomes
One funding flow contributes a larger share of resources compared to another	Service shifting: Healthcare providers could shift services from the funding flow that contributes less to a funding flow that contributes more to the overall resource envelope of the healthcare facilities to mobilize greater revenues.	**Inefficiency** because cost shifting leads to higher costs charged for services that could be paid for at a lower rate **Inefficiency** if service provision is shifted from a funding mechanism with a lower payment rate to a funding flow with a higher payment rate for the same service **Inefficiency** if resources are shifted to less cost-effective services **Inequity** if costs are shifted from a prepayment funding mechanism to an out-of-pocket mechanism **Inequity** in service use between patients that are discriminated, and those that are favoured Poor **quality of care** for patients that are discriminated against Poor **quality of care** for services that are underfunded due to resource shifting
	Resource shifting: Healthcare providers could shift resources away from services paid for by funding flows that contribute a small share of overall resources, to services that are paid for by funding flows that contribute large shares of overall resources to generate greater revenues. Healthcare providers could also discriminate against patients seeking services paid for by a funding flow that contributes to a small share of the overall resources.	
One funding flow is adequate to cover the cost of purchased services, while another funding source is inadequate	Cost shifting: Healthcare providers could shift costs to the funding flow that is adequate in covering the cost of purchased services.	
	Service shifting: Healthcare providers could shift service provision to the funding flow that is adequate in covering the cost of purchased services.	
	Resource shifting: Healthcare providers could shift resources away from services paid for by the funding flow that is inadequate, to services that are paid for by the funding flow that is adequate in covering the costs of services purchased. They could also favour patients seeking services that are paid for by a funding flow that is highly adequate (often better-off people) in covering the cost of services purchased.	
Healthcare providers have more flexibility over the use of one funding flow, compared to another	Cost shifting and service shifting: Healthcare providers could shift costs and/or services to the funding flow that healthcare providers have more flexibility over.	
	Resource shifting: Healthcare providers could shift resources away from services paid for by funding flows that are inflexible, to services that are paid for by funding flows that are more flexible. They could also discriminate against patients seeking services that are paid for by funding flows that healthcare providers have limited flexibility over their use.	
One funding flow has more complex and/or burdensome accountability requirements compared to another	Cost shifting and service shifting: Healthcare providers could shift costs and/or services to the funding flow that has less complex and/or burdensome accountability requirements.	
	Resource shifting: Healthcare providers could discriminate against patients seeking services that are paid for by a funding flow that has complex/burdensome accountability requirements.	
One funding flow is more predictable in terms of amounts and timeliness compared to another	Cost shifting and service shifting: Healthcare providers could shift costs and/or services to the funding flow that is more predictable.	
	Resource shifting: Healthcare providers could shift resources away from services paid for by funding flows that are less predictable, to services that are paid for by funding flows that are more predictable. They could also discriminate against patients seeking services that are paid for by a funding flow that is less predictable.	
One funding flow is linked to performance while another is not	Resource shifting: Healthcare providers could shift resources away from (or to) services paid for by funding flows that are linked to performance. They could also discriminate against (or in favour) of patients seeking services that are paid for by a funding flow that is linked to performance.	

Resource shifting could influence the equity, quality and efficiency in a health system. Resource shifting results in a redistribution of benefits such that one patient or a patient group benefits disproportionately from healthcare financing compared to another patient or a patient group. This favouring of certain patients that often belong to better-off population groups results in discrimination of those without a favourable funding flow (also referred to as patient cream skimming). When this disproportionate benefit is not due to need or in line with health service priorities, it introduces inequity in access of healthcare, i.e., it leads to discrimination among different groups of patients who go to the same healthcare provider. Resource shifting may create or aggravate under-resourced services and thus compromise the quality of care of service delivery. Resource shifting could also affect efficiency if resources are moved from more to less cost-effective services.

Service shifting could influence health system goals in several ways. For instance, when services are shifted from a prepaid funding flow to an out-of-pocket payment mechanism (e.g. through informal payments or balance billing), it could have equity implications. Likewise, it affects equity if the service shifting leads to additional and hence higher cost sharing or out-of-pocket expenditure. Service shifting from a funding flow with a lower provider payment rate to one with a higher payment rate for the same services has efficiency implications, because unnecessarily greater resources are used to achieve arguably similar outcomes. Further, quality of care could be compromised by providing unnecessary or harmful care, when service shifting involves over-provision.

Finally, cost shifting influences health system objectives, because when healthcare providers charge higher rates to different purchasers for the same service, both efficiency and equity may be compromised.

However, multiple funding flows, under the condition that they are coherent and aligned, can also incentivise favourable provider behaviour. For example, resource shifting can be desirable when healthcare providers put more attention on high priority services. This is the idea and objective of performance-based financing, in that healthcare providers receive an additional payment and are paid more when they reach set targets, such as higher case numbers for pre-determined priority services (Meessen *et al.*, 2015; Soucat *et al.*, 2017). When patient costs are shifted from out-of-pocket payments to prepayment funding flows, it could promote financial protection. Likewise, when cost shifting takes the form of charging higher rates to a funding flow used by the well-off (e.g. voluntary health insurance) to subsidize a funding flow used by the worse-off (e.g. a specific health coverage scheme for the poor or patients paying for services out-of-pocket), then equity could be improved.

### Applying the conceptual framework to assess a country’s multiple funding flows and related methodological challenges

To assess multiple funding flows, their attributes and potential effects on provider behaviour, we recommend a mixed methods approach by collecting both quantitative and qualitative data (Gilson *et al.*, 2011; Gilson, 2012). Qualitative data should entail document reviews and conducting interviews with purchasers and healthcare providers. Since health systems governance is crucial for strategic purchasing to occur, information on governance related aspects can also be collected through discussions with health system stewards, such as the ministry of health and other ministries or oversight board representatives. Focus group discussions could be conducted with community members to determine patient experiences about provider behaviour. Quantitative data on utilization rates, purchaser claims and payment data can serve to assess the effects of multiple funding flows on health system goals. In line with Figure 1, a stepwise assessment is proposed: (1) mapping purchasers, healthcare providers and funding flows; (2) analysis of funding flow attributes and related incentives; (3) exploration of provider behaviour and impacts on health system objectives (see also Mathauer and Dkhimi, 2019). Nonetheless, we acknowledge that it is difficult to measure precisely the effects of attributes and the resulting incentives and to quantify provider behaviour or impacts on health system objectives. An entry point is to identify indications pointing to the existence of a particular provider behaviour or indicating that there is a risk that undesirable provider behaviour may exist; possible indications are proposed in Mathauer and Dkhimi (2019). More methodological work will be needed on how to rigorously assess provider behaviour, measuring the effects of attributes of multiple funding flows and related incentives on provider behaviour and the impact on health system objectives. Even where there is variation in funding flow patterns to exploit in a statistical analysis, problems of selection and casemix differences among providers and funding flows will make it difficult to isolate the effects of the financial incentives.

## Discussion: policy options for managing multiple funding flows

The existence of multiple funding flows is often the result of a fragmented health financing system and a lack of governance to address this fragmented architecture and its consequences. Fragmentation can have many causes and is often intensified in decentralized government arrangements (Mathauer *et al.*, 2019c), which strengthens local decision-making but may also blur the visibility of funding flows to healthcare providers (Vilcu *et al.*, 2019). Here, governance is understood as ‘ensuring strategic policy frameworks exist and are combined with effective oversight, coalition-building, regulation, attention to system-design and accountability’ (WHO, 2007). It is an overarching health system function, which is of particular relevance for purchasing to be strategic (WHO, 2019). Related governance arrangements refer to institutional, legal and regulatory provisions through which oversight, guidance, regulation, as well as accountability of healthcare providers and purchasers, are exerted and through which harmonization and coordination across purchasers at system level are affected (WHO, 2019).

To reduce negative effects of multiple funding flows, healthcare providers must receive a coherent set of incentives. Coherence is taken here to mean that funding flows are aligned in such a way that the set of incentives results in desirable provider behaviours that promote rather than undermine the health system objectives of equity, quality, efficiency and financial protection. What are the policy options that policy makers can pursue to structure their purchasing arrangements in order to enhance the coherence of funding flows and mitigate against the undesired outcomes of such arrangements? Broadly, three strategies that relate to the governance of purchasing at different levels of the health system are required.

First, as part of governance of the overall healthcare purchasing system, health system stewards should reduce the fragmentation of purchasing arrangements and implement reforms to consolidate risk pools. Such reforms would lower the multiplicity of funding flows to healthcare providers and hence reduce incoherent signals to them (Mathauer *et al.*, 2020). However, it is not always feasible or desirable to consolidate risk pools for structural and political reasons. Further, even within single pools, multiple funding flows often exist.

As a second strategy, where multiple purchasers and attendant multiple funding flows persist as is often the case, health system stewards should seek to harmonize the attributes and hence the signals sent to healthcare providers in order to reduce or avoid incoherent incentives from different purchasers. For instance, provider payment rates could be harmonized such that healthcare providers do not get paid different rates for the same service by different purchasers and for different population groups (Mathauer *et al.*, 2020). Moreover, all funding flows to healthcare facilities should be subject to harmonized accountability and reporting requirements and decision space over their use. Likewise, each funding flow should be predictable with respect to disbursement and also adequate and sufficient to cover the costs of services purchased. Such harmonization will blunt provider responses by inhibiting the generation of negative incentives.

A third strategy is to constrain healthcare providers from responding to multiple funding flows in undesired ways. Here, effective governance arrangements targeting the healthcare provider level are required. This includes regulation related to when and how resource-, cost- and service-shifting is allowed, and monitoring and enforcement to prohibit undesired behaviour. Moreover, bottom-up accountability mechanisms such as patient and citizen feedback should also be strengthened by ensuring that they exist and are functional, and that feedback and complaints are acted upon. Some of the funding flow attributes themselves can be considered as governance arrangements to control healthcare providers. Accountability arrangements, for example, can be enhanced through supervision and control by the facility oversight committee, purchaser(s) and ultimately the ministry of health, as well as through reporting to these actors. Performance requirements can be outlined in explicit contracts between purchasers and healthcare providers and signal to healthcare providers which quality and quantity aspects need specific attention. Likewise, the degree of control granted to a healthcare provider over financing (financial autonomy) is an important instrument of provider level governance. Financial autonomy determines the level of flexibility that health care providers have over their funding flows and hence is decisive in influencing how healthcare providers react to the set of signals. Lastly, priority setting and resource allocation criteria at the healthcare provider level can promote more conducive resource shifting across services, patient needs and patient groups. When these governance arrangements are weak or absent, they make undesirable provider behaviour more likely.

## Conclusion

We have presented a conceptual framework for examining multiple funding flows that hypothesizes that preferences for certain attributes of a funding flow determine the dominant set of incentives that send signals to healthcare providers. These signals, in turn, influence provider behaviour, in ways which can be both desirable and undesired. The resulting provider behaviour then contributes to the achievement of equity, quality, efficiency and financial protection in healthcare service delivery positively or negatively. We have argued for the importance of taking a healthcare provider perspective, and for seeking to understand the set of incentives created by these multiple funding flows, rather than examining any one in isolation.

To address the challenges created by multiple funding flows, governance arrangements are critical. This is because the three proposed strategies for mitigating against the undesired effects of multiple funding flows (lowering the number of multiple purchasers, harmonizing signals from multiple funding flows, constraining healthcare providers to respond to undesirable incentives), as well as the political and institutional feasibility of these reforms are contingent upon effective governance arrangements.

Additional country studies on multiple funding flows will build the evidence and contribute to further develop the framework. Future research could also explore effects of incentives on the behaviour of individual health workers. Ultimately, more attention by policy makers and practitioners to align multiple funding flows will be an important step to better achieve health system objectives and to progress towards universal health coverage.
